# A Proposal of Cognitive Intervention in Patients with Alzheimer’s Disease through an Assembling Game: A Pilot Study

**DOI:** 10.3390/jcm11133907

**Published:** 2022-07-05

**Authors:** Remedios Navarro-Martos, Francisco Nieto-Escamez

**Affiliations:** 1 Departamento de Psicología, Universidad de Almería, Carretera del Sacramento S/N, 04120 Almería, Spain; remenavarro129@gmail.com; 2 Centro de Evaluación y Rehabilitación Neuropsicológica (CERNEP), Universidad de Almería, Carretera del Sacramento S/N, 04120 Almería, Spain

**Keywords:** dementia, Alzheimer’s disease, cognitive rehabilitation, non-pharmacological intervention, assembling game, executive function

## Abstract

(1) Background: Alzheimer’s disease is an irreversible brain disease, and current treatments are aimed at fighting cognitive decline. We have explored the feasibility of a game-based intervention for people with moderate Alzheimer’s disease; (2) Methods: Six participants, five women and one man, were recruited from a day center to participate in a five-week study, which included a re- and post-evaluation with the Montreal Cognitive Assessment (MoCA) and INECO Frontal Screening (IFS) tests. Three participants were assigned to the control group and three others to the experimental one. Both groups performed a play activity consisting of assembling a pie toy. Participants in the experimental group were asked to make a pie matching a sample after a time interval. Control group participants were asked to freely assemble a pie without the matching component; (3) Results: Patients were shown to be motivated during such an activity during the nine sessions. The experimental group showed a significant increase in IFS scores when comparing the post- and pre-intervention assessments. No significant differences were observed in MoCA scores; (4) The intervention created a social and emotional climate suitable to maintaining participants’ satisfaction and motivation, as well as to developing executive function while promoting positive emotions.

## 1. Introduction

Alheimer’s disease (AD) is considered the most common type of dementia, and its prevalence is increasing among the older population. AD is a multifactorial neurodegenerative disease characterized by progressive impairment in cognition, emotion, language and memory. Thus, AD patients can be classified as having preclinical AD, mild cognitive impairment (MCI), and mild, moderate or severe dementia along the disease continuum [1].

Early detection and diagnosis of AD can lead to earlier interventions, both pharmacological and non-pharmacological, to help maintain and improve physical and cognitive functioning [2]. The overall goals of treatment for AD are to maintain the quality of life; maximize function in daily activities; enhance cognition, mood and behavior; foster a safe environment; and promote social engagement. This involves regular monitoring of health and cognition in patients with AD, providing training and support to patients and their caregivers, and initiating drug and non-drug interventions [1].

The U.S. Food and Drug Administration (FDA) has approved six drugs for the treatment of AD. Five of these drugs—donepezil, rivastigmine, galantamine, memantine and memantine plus donepezil—temporarily alleviate AD symptoms but do not change the underlying brain pathology or change the course of the disease [1]. On the other hand, non-pharmacological strategies do not tackle the underlying biology of the disease and are aimed at training cognitive function, overall quality of life and engagement, and the ability to perform activities of daily living. Non-pharmacological treatments include cognitive stimulation, sensory stimulation and psychological treatment.

Non-pharmacological treatments to improve cognitive, behavioral and psychological symptoms in AD have received substantial attention over the last years. In particular, brain gaming has been reported as a promising intervention tool for different domains of dementia. Sood et al. [3] showed that computerized games are safe, user friendly and potentially effective to maintain or improve cognitive functions in adults with cognitive impairments. According to McCallum & Boletsis [4], the use of videogames in patients with neurodegenerative diseases can promote cognitive functions such as attention, memory, and visuospatial skills, and have a positive impact on the social and emotional life of patients, increasing sociability and reducing depression. However, most computerized tasks do not provide any kind of social interaction, and often, activities are not ecological and patients do not feel comfortable using technology. As an alternative, playing board games can be a useful strategy, as some authors have reported their protective role against dementia [5,6,7]. Moreover, these activities can be undertaken by elderly people suffering a physical disability, irrespective of the season or the weather, and can involve caregivers, family members or friends. Hughes et al. [8] found that an engagement in crossword puzzles and crafts reduced the risk of dementia independently from other activities, perhaps because these activities required more cognitive effort than other types of activities.

The purpose of this pilot study was to evaluate the feasibility of a board game-based intervention for cognitive rehabilitation in AD. The objectives of the intervention were: (i) to maintain participants’ motivation and self-esteem; (ii) to stimulate short-term memory; (iii) to stimulate other cognitive areas such as executive function; and (iv) to improve fine motor skills and visuospatial coordination. In short, it is expected that the intervention: (i) will show a good acceptance by the patients; (ii) will enhance fine motor and visuospatial skills; (iii) and that participants receiving cognitive training will improve their scores in the Montreal Cognitive Assessment (MoCA) and INECO Frontal Screening (IFS) tests.

## 2. Materials and Methods

### 2.1. Participants

Six participants of Spanish nationality were selected from Neuromar Day and Therapy Center (Roquetas de Mar, Spain). All the participants had been diagnosed with AD and scored between four and five on the Global Deterioration Scale. The participants were randomly allocated to the control or the experimental group, each one consisting of three participants. The control group consisted of two female participants and one male, aged between 76 and 84 years, while the experimental group was formed by three female participants aged between 77 and 86 years. All the participants and their legally responsible relatives were informed about the research characteristics and gave their informed consent before participation. None of the participants received monetary compensation. Table 1 describes participants’ characteristics.

### 2.2. Instruments

Two validated tests, the Spanish versions of the Montreal Cognitive Assessment test (MoCA) [11] and IFS [12], were used to assess participants’ cognition. The MoCA test assesses different cognitive abilities in patients with cognitive impairment, while the IFS evaluates executive functions.

As a measure of participants’ behavior, some data were registered during the sessions: participants’ verbalizations about the task, the number of dropouts (leaving the room before the session had ended), complaints about the activity (negative comments), refusals to participate (not wanting to carry out the activity) and signs of distraction (not paying attention, showing disinterest or a passive attitude). The frequency of these behaviors was recorded in every session.

**Figure 1:**
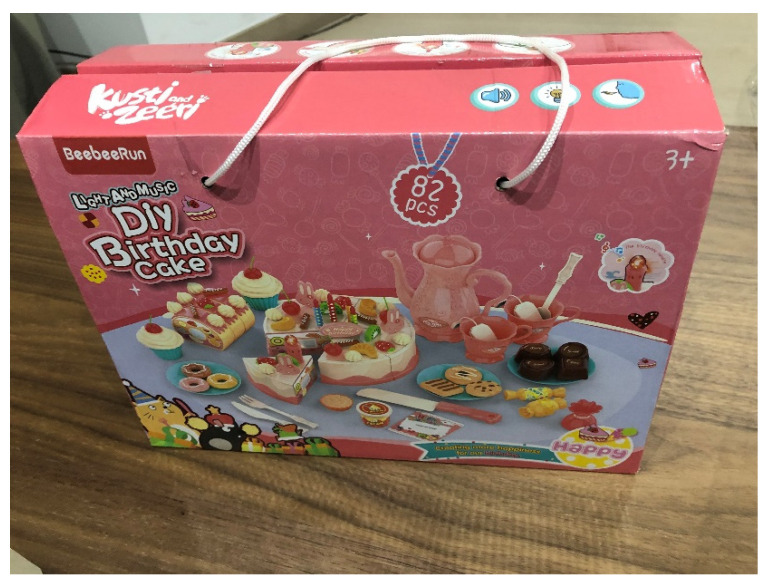
This picture shows a box containing the pie toy used in the intervention.

Lastly, as intervention material, we used a total of 6 configurable plastic pies (BeebeeRun Diy Birthday cake, Plastic Toys Co., Ltd., Shantou, China). Some parts were assembled and used as sample models (one per participant), and another set of parts were given to the participants to be assembled so as to match the model shown immediately before. Each pie has a total of 82 possible parts, including up to 6 pie slices that could be assembled and decorated with different accessories, including a coffee set, chocolates, candies, muffins… (see Figure 1).

### 2.3. Procedure

The study lasted five weeks and was carried out in the Day Center that the participants attended daily. It consisted of four phases: (1) Pre-assessment of participants’ cognitive capabilities, which took four days. The MoCA test was administered to all the participants during the first two days, and then the IFS test was administered for another two days. (2) Subsequently, all the participants were allowed to handle and play with the intervention material (pie parts and accessories) for another two sessions. Participants handled and decorated the pie slices (originally a bare plastic part without any picture), attaching stickers onto the pie slice so it looked like real food. Then, participants spent time making pies to their liking. (3) Subsequently, the intervention phase was carried out for three weeks, three days per week, with a duration of about 45 min per session. In total, nine sessions were carried out. (4) The study ended with the post-assessment of cognitive variables after the intervention phase (same protocol as in the pre-assessment phase).

**Figure 2:**
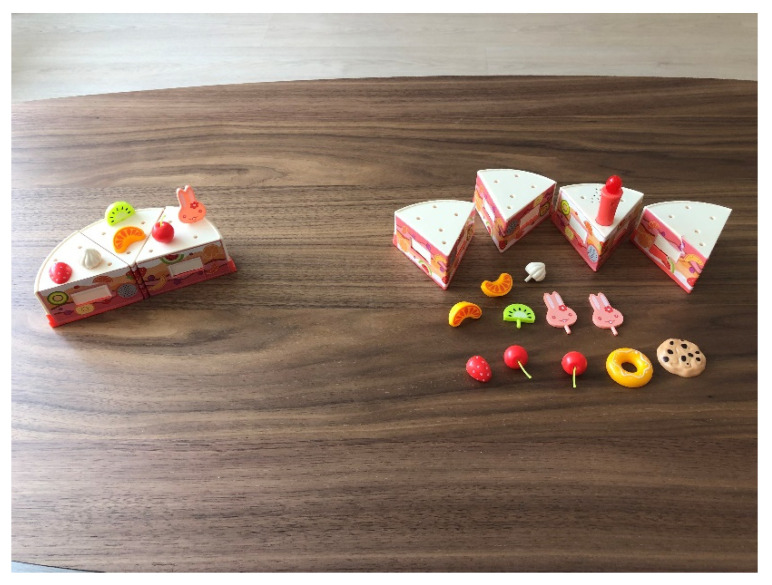
On the left, a sample pie as shown to the participants by the researcher. On the right, the parts that the participant received to match the sample pie.

During the nine intervention sessions, each participant had to assemble a plastic pie. Participants allocated to the control group were allowed to play freely with different parts, assembling pie slices and decorating them to their liking. Participants allocated to the experimental group had to assemble their pie so as to match a model that the researcher had previously shown (See Figure 2). A different pie model was used in every session. The intervention consisted in the researcher initially assembling a pie model in front of the participant, describing—verbally and with gestures—which parts were used and where they should be placed. Then, the pie model assembled by the researcher was hidden, and the participant was asked to make a pie with the same appearance. At the beginning of every session, each participant received 15 parts and needed to select those required to build the pie. These parts were placed in front of the participant at the beginning of the session. In each session, the parts were different, like the pie model that had to be made. The three participants sat side by side at a large table, and whenever the researcher interacted with a participant, she sat in front of her. All the procedures were carried out in the same room, a quiet environment that the participants were used to.

Each pie model was different for each participant. With regards to the control group, participants were free to make their own pie without having to match a model shown before. The time for handling pie parts was the same for the control and experimental groups.

Verbal and physical rewards were used. Participants received compliments from the researcher in all the sessions during the activity (emphasizing good choices), as well as after completing the task. The researcher used expressions such as “well done!” and “you are making a beautiful pie!” when the participant was assembling her pie, or “Congratulations, you have made it!”. Participants were also rewarded with origami figures with different themes (flowers, birds or heart-shaped envelopes) at the end of each session.

The complexity of the task increased gradually in subsequent sessions by adding more pieces to the pie model. In the first week, nine parts were used, in the second week, 11 parts, and in the third week, 13 parts. Thus, participants had to pay attention to and progressively store more items in their short-term memory. The level of difficulty was adapted to the cognitive capabilities and performance of the participants. In case the task was not performed correctly, the researcher provided help at the end of the task, showing the model again for a few seconds and pointing out the specific part in which difficulties were observed. Then, the participant was asked to fix the mistakes and continue assembling the pie until it was like the model.

### 2.4. Assessment

A researcher different to the fellow responsible for the intervention was in charge of assessing participants’ cognitive function in the pre- and post-intervention assessment phases, both for the MoCA and IFS questionnaires. Furthermore, participants’ behavior was registered by the researcher who conducted the intervention. A record registered participants’ positive verbalizations regarding the researcher, the task or the rewards, dropouts (get up from the chair and leave the room), complaints regarding the researcher, the task or the rewards, and distractions. This record provided information about participants’ motivation for the task performance. Orientation, visuospatial processing and fine motor skills were also assessed with the corresponding MoCA and IFS subscales. A comparison between pre- and post-intervention total scores in MoCA and IFS was done for the experimental group, and a comparison between the control and experimental groups was also done.

### 2.5. Analysis

Quantitative data were analyzed by using the software SPSS 27. Parametric paired *t*-tests were used to compare the pre- and post-intervention performance of the experimental group, both for IFS and MoCA scores. In both cases, the Shapiro–Wilk test of normality was conducted to check that the distribution of the sample was normal. A non-parametric Mann–Whitney U test was used to compare the control and experimental group scores in the post-intervention assessment, both for IFS and MoCA tests.

## 3. Results

Regarding participants’ behavior, there were no dropouts, and all the participants showed interest in the activity.

Regarding the cognitive results, the Shapiro–Wilk test showed a normal distribution for pre- and post-intervention scores of the experimental group for IFS and MoCA tests (*p* > 0.05). The paired *t*-test reported significant differences in the experimental group when the IFS total score of the pre-intervention assessment (Mean = 11.00, SD = 2.65) was compared with the post-intervention assessment (Mean = 15.83, SD = 3.75, t(2) = 5.8, *p* = 0.028, d = 3.349). However, no significant differences between pre- and post-intervention scores were observed for any particular subtest of IFS. However, the Mann–Whitney U test did not show significant differences between control and experimental groups for the total IFS score (U = 2, *p* = 0.268) or for any IFS subtest in the post-intervention assessment.

The comparison between pre-intervention (Mean = 8.33, SD = 2.89) and post-intervention (Mean = 10.00, SD = 3.00) for MoCA total scores in the experimental group did not reach statistical significance when a paired t-test was performed (t(2) = 0.508, *p* = 0.66), nor did it do so for any MoCA subtest.

## 4. Discussion

In the present work, we have tested the suitability of a game requiring AD patients to build a pie mock-up that matched a previously shown model. According to participants’ behavior and verbalizations during the activity, we can affirm that the task was positively accepted by AD patients. In general, participants made positive verbalizations and referred to positive feelings after completing the activity, particularly those allocated to the experimental group (e.g., “this game is very fun and remembers me when I was a child”, “I like it”, “I’ve done it well”). They also expressed gratitude to the researcher (e.g., “You are very patient with me and I’m having fun”). Participants showed positive behaviors like clapping or smiling when the pie was completed or proudly displayed their pie to the researcher and the other participants. In general, there were very few complaints and signs of disinterest, which were expressed by participants allocated to the control group, mainly by the male participant who considered that the activity was a feminine task. It is essential for the activity to be attractive and fun in order to keep participants’ attention and motivation [13]. This is important to facilitate the generalization of the benefits of the training, and therefore the intervention must tackle cognitive training among other factors such as motivation and emotion. Among the factors that would have influenced such a good acceptance are the use of different types of rewards, verbal and physical, during and at the end of the activity. Additionally, during the performance of the activity the participants received continuous feedback and the researcher helped them when they expressed any difficulty. Ruiz–Sanchez et al. [14] have stated that the use of a methodology of learning without error together with reinforcements at the end of the task have been shown to be beneficial, not only to restore cognitive functions but also to increase patients’ self-esteem and motivation.

Finally, contrary to many gaming activities that patients perform alone, the activity was carried out as part of a group activity but was adapted to the characteristics of each participant. This serves to increase patients’ motivation and their satisfaction with the activity. Therefore, our results are in line with other studies showing that AD patients positively rate cognitive training through serious games, resulting in high levels of satisfaction and positive emotions towards the activity [15].

The comparison between the pre- and post-intervention assessment of participants in the MoCA did not reveal significant differences (although both groups increased their global scores). The absence of significant differences between both assessment times was observed in all the subtests and in the global score of this instrument. This could indicate that the intervention was not effective in improving participants’ cognition. However, IFS scores in the experimental group showed a significant improvement after the intervention phase. The IFS questionnaire provides a measure of executive functioning and is made of different subtests that assess three different domains (response inhibition and set shifting, abstraction, and working memory). Individually, none of the IFS subtests showed significant differences between pre- and post-intervention assessment scores. Such a result would mean that the intervention did not increase the performance in any particular domain of the instrument, but the observed improvement was the consequence of an additive effect of all the measures making up the IFS. To the best of our knowledge, there are no cases in the literature reporting an improvement of executive functions after training AD patients with a game similar to the one employed in the present study. Recently, Zhang et al. [16] reported that playing Mahjong for 12 weeks improved the executive function of elderly people with Mild Cognitive Impairment. Similarly, Kuo et al. [17] found that multi-component cognitive training by playing card games for eight weeks improved executive functions in old adults, in contrast to an active control group. We must remark that assembling a pie matching a sample shown before can also be considered a multi-component procedure, as it consists in a social engagement activity that requires a visual search, provides training for eye-hand coordination, and participants must pay attention to the parts and visuospatial features of the pie and maintain such information in their working memory. It also involved inhibitory control, so as to use only those parts that were present in the sample and switch when any part was wrong or incorrectly assembled.

Therefore, board games can be considered a suitable strategy for improving executive functions, not just in elderly but also in dementia patients, although the characteristics of the game are a factor to consider. Moreover, differences observed between the MoCA and IFS results would indicate that these tests would have a different sensitivity in detecting the improvement of executive function in AD patients. 

It has been previously reported that moderately-to-severely demented AD patients can learn and retain fine motor skills and maintain implicit motor skills [18,19]. It has also been reported that memory improves after a cognitive rehabilitation program in AD patients [20]. However, a recent meta-analysis by Kletzel et al. [21]. reported that cognitive rehabilitation through brain gaming did not produce effects on the cognitive domains of memory, executive function, visuospatial skills, and language. Our study did not obtain significant improvements in any MoCA or IFS subtests, including motor, response inhibition, memory, visuospatial, attention, language, abstraction or orientation functions. It is probable that in order to achieve a significant improvement in any of these functions, a longer intervention is required. 

We must acknowledge some limitations in the present study. First, the small size of the sample impedes the generalization of the present results. Second, the short duration of the intervention (only nine sessions). Third, only two validated instruments, the MoCA and IFS tests, were employed. Additional and specific tests should be included in further research. And fourth, the post-training assessment was conducted after the intervention. It would be relevant to carry out a follow-up assessment to investigate if the effect is maintained in the medium and long terms. Moreover, complaints from the male participant mentioning the feminine nature of the activity point to the need to check if participants have any prejudice against the activity. This work was conceived as a pilot study aimed at assessing the feasibility of the procedure. However, the observed results were partially positive and reached statistical significance with a relevant effect size. This encourages us to continue our research, carrying out in the near future a controlled randomized trial based on this training procedure. This will increase the objectivity and generalizability of these preliminary results.

## 5. Conclusions

The present pilot study demonstrates the feasibility of our intervention procedure as a strategy to improve cognitive functioning in moderate cases of AD. Patients showed a willingness to do the activity and judged it positively both during and after its execution. Moreover, despite the reduced number of participants and the short duration of the intervention, a significant improvement in the IFS total score was observed in the post-intervention assessment. Nevertheless, we must acknowledge that the reduced sample of participants and the limited number of analyzed variables do not permit one to generalize the results obtained in the current work. This encourages us to continue our research in a future, controlled work that will use a larger sample and more sensitive assessment tools. 

## Figures and Tables

**Table 1:** Participants’ characteristics.

Participant	Sex	Group	Age	Education	MMSE *	GDS	Medication
1	Female	Experimental	86	Primary (literate)	20/35	GDS4	Donepezil 10 mg
2	Female	Experimental	78	Primary (literate)	18/30	GDS5	NO
3	Female	Experimental	77	Primary (literate)	15/30	GDS5	Memantine 20 mg
4	Female	Control	84	Primary (literate)	22/30	GDS4	Memantine 20 mg
5	Male	Control	81	Primary (literate)	12/35	GDS5	Memantine 20 mg
6	Female	Control	76	Primary (literate)	16/30	GDS5	Memantine 20 mg Donepezil 10 mg

* Spanish versions of MMSE, the MEC-30 [9] or the MEC-35 [10] were used.

## Data Availability

The data that support the findings of this study are available from the corresponding author upon reasonable request.
